# Delineating the pattern of treatment for elderly locally advanced NSCLC and predicting outcomes by a validated model: A SEER based analysis

**DOI:** 10.1002/cam4.2127

**Published:** 2019-04-03

**Authors:** Meiying Guo, Butuo Li, Yishan Yu, Shijiang Wang, Yiyue Xu, Xindong Sun, Linlin Wang, Jinming Yu

**Affiliations:** ^1^ School of Medicine Shandong University Jinan China; ^2^ Department of Radiation Oncology Shandong Cancer Hospital Affiliated to Shandong University Jinan China; ^3^ Department of Radiation Oncology and Key Laboratory of Cancer Prevention and Therapy, Tianjin Medical University Cancer Institute and Hospital National Clinical Research Center for Cancer Tianjin China; ^4^ Shandong Academy of Medical Sciences Jinan China

**Keywords:** CSS, Geriatric, Locally advanced nonsmall‐cell lung cancer, nomogram, OS, SEER

## Abstract

**Introduction:**

Locally advanced nonsmall‐cell lung cancer (LA‐NSCLC) represented a highly heterogeneous group, with more than half of the patients aged ≥65 years at the time of diagnosis. However, the optimal treatment for elderly LA‐NSCLC patients was still not defined.

**Methods:**

A total of 33530 elderly patients (≥65 years) diagnosed with LA‐NSCLC from 2004 to 2014 were identified from Surveillance, Epidemiology, and End Results (SEER) database.

**Results:**

Locally advanced nonsmall‐cell lung cancer patients aged 65‐74 years were more frequently treated with chemoradiotherapy (CRT) (40%), while patients aged ≥75 years received more best supportive care (BSC) (36%). For age group of 65‐74 years, patients who had surgery with or without (neo)adjuvant therapy had a median survival of 28 months, CRT 15 months, radiotherapy (RT) alone 6 months, chemotherapy alone 11 months, and BSC 3 months; while for patients aged ≥ 75 years, the median OS was 20, 13, 7, 9, and 2, respectively. Besides, independent clinicopathological factors were integrated into nomograms for OS and CSS prediction, C‐indexes achieved 0.692 and 0.698, respectively. Importantly, the discrimination of nomogram was superior to that of the American Joint Committee on Cancer TNM classification (0.742 vs 0.572 for training set and 0.731 vs 0.565 for validation set).

**Conclusion:**

For elderly patients with LA‐NSCLC, the curative‐intent treatment (surgery or CRT) conferred better survival compared to chemotherapy alone, RT alone and BSC. The proposed nomograms based on independent clinicopathological variables may be practical and helpful for precise evaluation of patient prognosis, and guiding the individualized treatment for elderly LA‐NSCLC.

## 1 INTRODUCTION

Lung cancer remains the leading cause of death worldwide, and ~ 30% of nonsmall‐cell lung cancer (NSCLC) patients are diagnosed with locally advanced disease (LA‐NSCLC).^1^ Of those, nearly two‐thirds of patients are aged ≥65 years at the time of diagnosis,^2^ demonstrating a large proportion of this patient population is elderly. However, the elder patients were always underrepresented in the prospective clinical trials, resulting in a relatively little information guiding the clinical decision.^3^ Therefore, the study on the delineating of the treatment pattern during the real‐world clinical practice and its prognostic role in patient outcomes was particularly important for elderly LA‐NSCLC.

For relatively fit and younger patients, surgery with adjuvant chemotherapy or radiotherapy (RT) is considered standard treatment for resectable LA‐NSCLC; and concurrent chemoradiotherapy (CRT) is recommended for patients with unresectable disease.^4^, ^5^, ^6^ However, these data seemed not to be simply extrapolated to the elderly patients due to their poor characteristics of worse performance status (PS), reduced hepatic and renal function, and more comorbidities.^7^, ^8^ Surgery was reported to be associated with more morbidity and postoperative mortality for patients with >70 years^9^; and the survival was not found to be significantly superior for the intensive therapy with concurrent CRT compared to sequential CRT or RT alone.^10^ Thus, less aggressive treatment or the palliative therapy was more likely to be chosen for elderly LA‐NSCLC.^11^ Nevertheless, a recent research showed that age alone was not sufficient to be determined for omitting aggressive treatment,^12^ and the elder patients received curative therapy including surgery were found to have similar survival with the younger.^13^, ^14^ Among patients aged with 65‐74 years, surgery and CRT could reduce the hazard ratio (HR) of death compared to the RT or chemotherapy alone.^15^ Thus, the optimal approach in the elderly LA‐NSCLC remains a matter of debate, which needs to well balance the patient outcome and their ability to withstand the treatment.

Precise estimation of prognosis is crucial for guiding individualized treatment strategies. Although American Joint Committee on Cancer (AJCC) TNM staging system is commonly utilized for predicting prognosis of NSCLC, other clinicopathological features such as age at diagnosis, gender, histology, tumor site, tumor grade, and primary tumor size can also influence patient outcomes.^16^ Thus, the predictive model incorporated different prognostic clinicopathological factors may be more accurate and informative for prognosis evaluation and making clinical decision. Nomogram, an integrative prognostic model, has been found to facilitate better treatment stratification and outcome evaluation in breast cancer, gastrointestinal cancer, and prostate cancer.^17^, ^18^, ^19^ However, currently no nomogram has been established for predicting the survival of elderly patients with LA‐NSCLC.

Since the lack of evidence from the prospective clinical trials, we speculated that the population‐based analysis of elderly LA‐NSCLC would be more representative and informative. Thus, the objective of this study was to describe patterns of treatment and survival in elderly patients with LA‐NSCLC, and to develop nomograms for individualized predicting their prognosis.

## 2 METHODS

### 2.1 Data source and inclusion criteria

**Figure 1:**
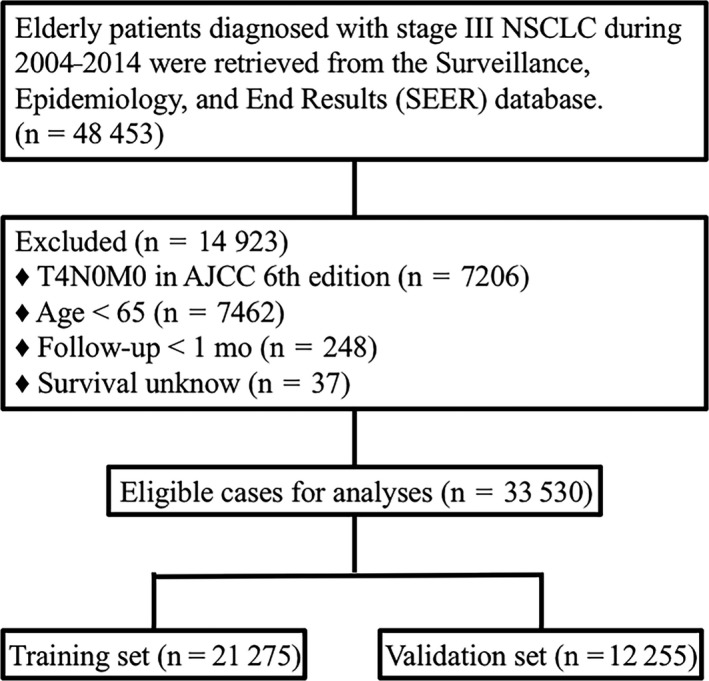
Flow diagram of eligible elderly patients diagnosed with locally advanced nonsmall‐cell lung cancer (2004‐2014). AJCC, American Joint Committee on Cancer; NSCLC, Nonsmall‐cell lung cancer

The Surveillance, Epidemiology, and End Results (SEER) database is a national collaboration program, which collects information of cancer patients in 18 registries, covering ~ 30% of total U.S population. Elderly patients diagnosed with LA‐NSCLC from 2004 to 2014 were identified from SEER database. SEER*Stat 8.3.5 software (National Cancer Institute, Bethesda, MD) was used to extract per‐patient data. The flow diagram of data selection is shown in Figure 1. Only patients who met the following criteria were included: (1) age ≥65 years; (2) diagnosed with stage III NSCLC as its first and only malignancy; (3) ICD‐O‐3 codes were limited to 8012 (large cell carcinoma), 8046 (nonsmall‐cell carcinoma), 8070 (squamous carcinoma), and 8140 (adenocarcinoma); (4) active follow‐up with complete date and known survival months and known cause of death. The patient cohort consisted of patients who used AJCC sixth and seventh staging criteria, as AJCC sixth edition updated in 2010. Patients with stage T4N0M0 in the AJCC sixth edition were excluded because these patients may be stage II (T3N0M0) or stage IV (M1a ‐ malignant pleural or pericardial effusion) in the AJCC seventh edition. In addition, follow‐up time less than 1 month was excluded from the analysis to limit immortal time bias.^20^

This study was approved by the institutional review board of Shandong Cancer Hospital Affiliated to Shandong University.

### 2.2 Study variables

A total of 33530 patients from 18 SEER registries were obtained, patients from four randomly selected registries (Louisiana, New Jersey, Georgia, Kentucky) were assigned as validation data set, and from other registries were regarded as training set. Demographic and clinical variables included age at diagnosis (65‐74 years and ≥ 75 years), gender (male and female), tumor site (upper lobe, middle lobe, lower lobe, main bronchus and overlapping lesion), tumor grade (well differentiated, moderately differentiated, poorly differentiated and undifferentiated), laterality (left and right), histology (squamous cell carcinoma, adenocarcinoma, large cell carcinoma and other NSCLC), stage (IIIA and IIIB), TN category, tumor size, and first‐line treatment. The treatment was categorized as surgery (including (neo)adjuvant therapy (if applicable)), CRT (including concurrent CRT and sequential CRT), RT alone (both curative‐intent and palliative), chemotherapy alone, and best supportive care (BSC). The primary outcomes of this study were overall survival (OS) and cause‐specific survival (CSS). OS was defined as the time from diagnosis to death from all possible causes and CSS was measured as the time from diagnosis to death attributed to NSCLC. Patients who were alive at the time of last follow‐up were counted as censored observations.

### 2.3 Statistical analyses

Clinicopathologic characteristics were evaluated for training set and validation set according to age groups, and statistically significant differences (*P* < 0.05 two sided) were determined by the chi squared test for categorical variables and the one‐way Analysis of Variance (ANOVA) for continuous variables. Survival curves were generated using Kaplan‐Meier estimates, and the differences were analyzed by log‐rank test. Both univariate and multivariate Cox proportional hazard regression models were used to explore the association between risk factors and survival outcomes. For the determination of the independent factors for OS and CSS, variables with *P* < 0.15 in univariate analyses were entered into multivariate analyses and analyzed with backward selection procedure. Results of the Cox regression modeling are presented as HRs and associated 95% confidence intervals (CIs). All statistical tests were two‐sided, and *P* < 0.05 were considered to be statistically significant. Statistical evaluation was conducted with SPSS 23.0 (SPSS Inc, Chicago, IL).

### 2.4 Nomogram

The model of nomogram based on the data of training set was constructed. According to the results of Cox proportional hazard regression, nomogram combining all the independent prognostic factors was established for predicting 2‐year and 5‐year OS or CSS by using the package of rms in R software version 3.4.4 (Institute for Statistics and Mathematics, Vienna, Austria). The performance of the nomogram was evaluated by discrimination and calibration using validation set. Concordance index (C‐index) values were used to measure discriminative ability, which is appropriate for censored data.^21^ C‐index ranges from 0.5 to 1.0, 0.5 means a random chance to correctly discriminate outcome with the model while 1.0 stands for a prefect matching. For the sake of calibration, the data were divided into several groups based on the probabilities calculated by the nomogram predictive model. Subsequently, predicted probabilities generated by the nomogram was compared with actual probabilities by the Kaplan‐Meier method. Chi‐square statistic and bootstrapping correction were used for this purpose. Furthermore, the prediction between the proposed nomogram and the seventh edition of AJCC staging system were performed by the area under receiver operating characteristic (ROC) curve (AUC). AUC was calculated separately from the first to the 60th month. Integrated AUC was considered to be their average. The difference between nomograms and TNM stage were compared with the method of DeLong et al (1988) using MedCalc Statistical Software version 18.2.1 (MedCalc Software bvba, Ostend, Belgium).^22^

## 3 RESULTS

### 3.1 Patients’ baseline characteristics

A total of 33530 elderly patients with LA‐NSCLC diagnosed between 2004 and 2014 were identified from SEER database. The proportion of patients aged 65‐74 years and ≥75 years were 51.3% and 48.7%, respectively. The demographic features and clinicopathological characteristics of the training set (n = 21275) and validation set (n = 12255) are presented in Table 1.

**Table 1:** The demographic and clinicopathological variables of the training set and validation set

	Training set (n = 21275)					Validation set (n = 12255)				
	65‐74		≥75		*P* value	65‐74		≥75		*P* value
	No.	%	No.	%		No.	%	No.	%	
Gender										
Male	5913	56.8	5650	52.0	<0.001	4040	59.3	2939	54.0	<0.001
Female	4495	43.2	5217	48.0		2771	40.7	2505	46.0	
Site										
Upper lobe	5802	61.1	5677	58.3	<0.001	2823	53.8	2886	59.4	<0.001
Middle lobe	366	3.9	411	4.2		273	5.2	200	4.1	
Lower lobe	2558	26.9	3052	31.3		1667	31.8	1453	29.9	
Main bronchus	611	6.4	479	4.9		402	7.7	253	5.2	
Overlapping lesion of lung	163	1.7	119	1.2		83	1.6	63	1.3	
Grade										
Well differentiated	219	3.9	280	5.1	0.013	130	3.5	125	4.7	0.029
Moderately differentiated	1777	31.8	1675	31.3		1177	32.0	815	30.6	
Poorly differentiated	3419	61.2	3243	60.5		2243	61.0	1648	61.9	
Undifferentiated	172	5.1	162	3.3		128	3.5	73	2.7	
Laterality										
Left	4159	40.7	4369	40.9	0.800	2682	39.9	2172	40.6	0.466
Right	6052	59.3	6311	59.1		4036	60.1	3180	59.4	
Histology										
Squamous cell carcinoma	3613	34.7	3669	33.8	0.086	2916	42.8	2174	39.9	0.008
Adenocarcinoma	4292	41.2	4440	40.9		2403	35.3	1983	36.4	
Large cell carcinoma	252	2.4	253	2.3		227	3.3	181	3.3	
Others	2251	21.6	2505	23.1		1265	18.6	1106	20.3	
T stage										
T1	1229	12.4	1045	10.1	<0.001	824	12.8	517	9.9	<0.001
T2	2883	29.1	2887	27.9		1974	30.6	1520	29.1	
T3	893	9.0	769	7.4		694	10.8	519	9.9	
T4	4886	49.4	5662	54.6		2962	45.9	2665	51.1	
N stage										
N0	1436	14.1	2025	19.3	<0.001	878	13.0	992	18.6	<0.001
N1	623	6.1	618	5.9		433	6.4	353	6.6	
N2	6502	63.9	6479	61.6		4408	65.5	3297	62.0	
N3	1618	15.9	1391	13.2		1011	15.0	679	12.8	
Stage										
IIIA	6348	61.0	6781	62.4	0.036	4286	62.9	3525	64.8	0.038
IIIB	4060	39.0	4086	37.6		2525	37.1	1919	35.2	
Tumor size	/	/	/	/	0.480	/	/	/	/	0.696
Treatment										
Surgery	1774	17.0	1040	9.6	<0.001	1064	15.6	490	9.0	<0.001
Chemoradiotherapy	4109	39.5	2563	23.6		2773	40.7	1397	25.7	
Radiotherapy	945	9.7	1813	16.7		758	11.1	1045	19.2	
Chemotherapy	1495	14.4	1445	13.3		854	12.5	663	12.2	
Best supportive care	2085	20.0	4006	36.9		1362	20.0	1849	34.0	

The *P* values were determined by the chi squared test for categorical variables and the one‐way ANOVA (Analysis of Variance) for continuous variables./, not applicable.

### 3.2 Patterns of treatment in elderly patients

**Figure 2:**
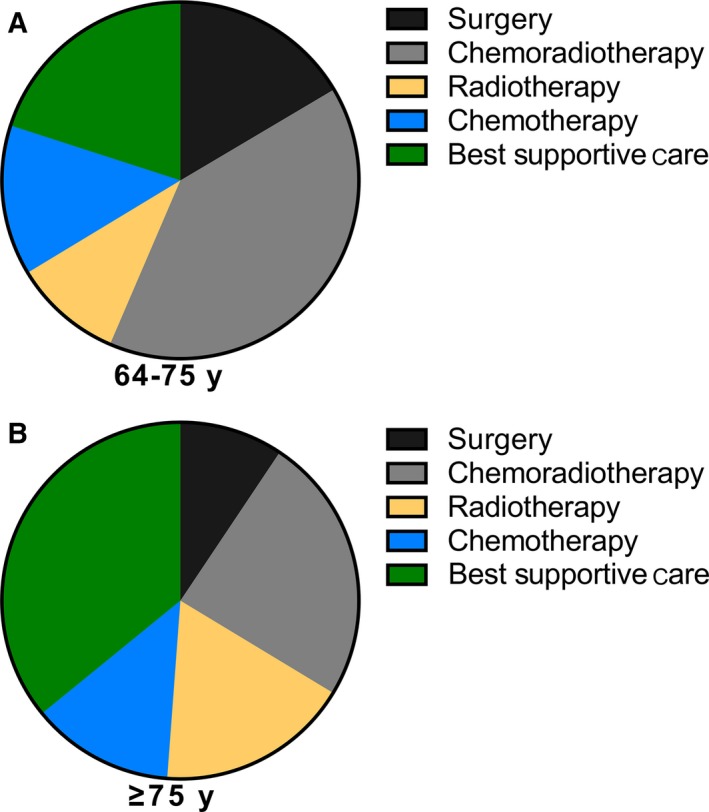
Distribution of treatment for elderly patients diagnosed with locally advanced nonsmall‐cell lung cancer. (A) Representing patients aged 65‐74 years; (B) representing patients aged ≥ 75 years

For the whole study population, the treatment of elderly patients with different age group were shown in Figure 2. The majority of patients aged 65‐74 years received CRT (40.0%), while BSC was the most wide choice for patients ≥75 years (35.9%). Compared to the ≥75 years age group, patients aged 65‐74 years more often underwent surgery (16% vs 9%), CRT (40% vs 24%), and received less RT (10% vs 18%) as well as BSC (20% vs 36%).

### 3.3 Prognosis of different treatments for elderly patients

Median OS was significantly shorter for all the enrolled patients aged ≥75 years compared with 65‐74 years (8.0 months vs 12.0 months, log‐rank *P* < 0.001). The respective 1‐, 2‐, and 5‐year OS rates were 47.8%, 28.1%, and 11.4% in patients aged 65‐74 years, and 35.0%, 18.2%, and 5.6% in patients aged ≥ 75 years (Figure S1).

**Figure 3:**
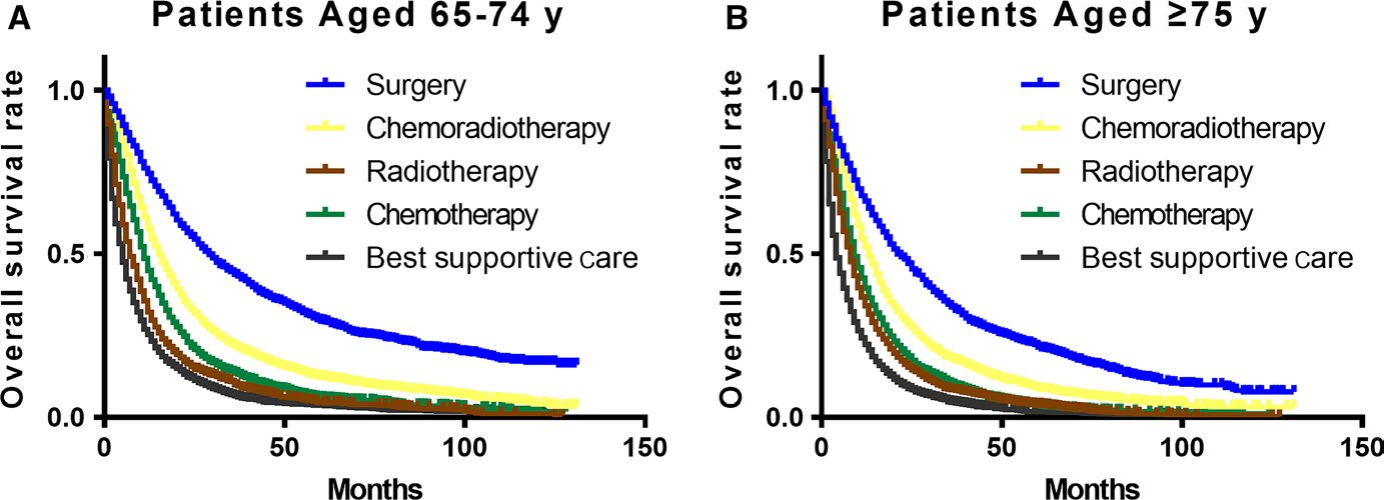
Overall survival of elderly patients diagnosed with locally advanced nonsmall‐cell lung cancer according to type of treatment. (A) Representing patients aged 65‐74 years, log‐rank *P* < 0.001; (B) representing patients aged ≥ 75 years, log‐rank *P* < 0.001. All curves represent actual survival evaluated by Kaplan‐Meier

For age group of 65‐74 years, patients who had surgery with or without (neo) adjuvant therapy had a median survival of 28 months, CRT 15 months, RT alone 6 months and chemotherapy alone 11 months. BSC resulted in a median OS of 3 months; while patients in aged ≥75 years, the median OS was 20, 13, 7, 9, and 2, respectively (Figure 3). Then, outcomes of risk of death adjusted for treatment were further analyzed (in Table S1). Elderly patients receiving surgery with or without (neo) adjuvant therapy had best prognosis in both the two age groups (both *P* < 0.001). The application of CRT showed an increased risk of death compared to surgery (HR 1.55 95% CI 1.47‐1.64 *P* < 0.001 and HR 1.27 95% CI 1.18‐1.37 *P* < 0.001, respectively for patients aged 65‐74 years and ≥75 years), but it significantly prolongs survival across all two age groups compared to RT alone (both *P* < 0.001) and chemotherapy alone (both *P* < 0.001). The highest risk of death was seen with BSC, which was more profound among patients aged 65‐74 years (HR 4.31 95% CI 4.04‐4.60) compared to those aged ≥75 years (HR 3.48 95% CI 3.23‐3.73).

### 3.4 Univariable and multivariable analysis for OS and CSS

For further exploring the independent factors for predicting patient outcomes, univariate and multivariate analyses using Cox proportional hazards regression modeling were firstly performed for patients in training set (n = 21,275). As shown in Table 2, age at diagnosis, gender, tumor site, tumor grade, laterality, histological type, AJCC TNM stage, T stage, N stage, tumor size, and treatment were remarkably correlated with OS and CSS in univariate survival analyses (*P* < 0.001). Variables with *P* < 0.15 in univariate analyses were entered into multivariate model and analyzed with backward selection procedure. In multivariate analysis, age at diagnosis, gender, tumor site, tumor grade, histological type, T stage, N stage, tumor size, and treatment were verified to be independent prognostic factors for OS. And except for the histological type, all other independent factors for OS were also significantly associated with CSS in multivariate analysis.

**Table 2:** Univariate and multivariate survival analyses of OS and CSS in elderly patients with LA‐NSCLC

	OS						**CSS**					
	Univariate analysis			Multivariate analysis			Univariate analysis			Multivariate analysis		
	HR	CI	*P* value	HR	CI	*P* value	HR	CI	*P* value	HR	CI	*P* value
Age												
65‐74	Ref		<0.001	Ref		<0.001	Ref		<0.001	Ref		<0.001
≥75	1.420	1.379‐1.462	<0.001	1.196	1.139‐1.256	<0.001	1.407	1.364‐1.452	<0.001	1.166	1.106‐1.228	<0.001
Gender												
Male	Ref		<0.001	Ref		<0.001	Ref		<0.001	Ref		<0.001
Female	0.881	0.856‐0.907	<0.001	0.866	0.826‐0.909	<0.001	0.898	0.870‐0.926	<0.001	0.887	0.843‐0.934	<0.001
Site												
Upper lobe	Ref		<0.001	Ref		<0.001	Ref		<0.001	Ref		<0.001
Middle lobe	1.074	0.992‐1.162	0.079	1.121	0.990‐1.270	0.073	1.097	1.009‐1.193	0.031	1.170	1.025‐1.336	0.020
Lower lobe	1.127	1.089‐1.167	<0.001	1.126	1.068‐1.188	<0.001	1.130	1.089‐1.173	<0.001	1.134	1.071‐1.201	<0.001
Main bronchus	1.462	1.369‐1.561	<0.001	1.308	1.169‐1.463	<0.001	1.485	1.385‐1.593	<0.001	1.326	1.178‐1.493	<0.001
Overlapping lesion of lung	1.204	1.060‐1.366	0.004	1.113	0.929‐1.334	0.245	1.200	1.047‐1.376	0.009	1.120	0.921‐1.361	0.257
Grade												
Well differentiated	Ref		<0.001	Ref		<0.001	Ref		<0.001	Ref		<0.001
Moderately differentiated	1.147	1.032‐1.276	0.011	1.316	1.162‐1.490	<0.001	1.148	1.023‐1.287	0.019	1.326	1.159‐1.516	<0.001
Poorly differentiated	1.346	1.214‐1.492	<0.001	1.388	1.228‐1.568	<0.001	1.374	1.229‐1.536	<0.001	1.416	1.240‐1.616	<0.001
Undifferentiated	1.587	1.364‐1.846	<0.001	1.575	1.302‐1.906	<0.001	1.633	1.389‐1.920	<0.001	1.596	1.301‐1.959	<0.001
Laterality												
Left	Ref		0.143				Ref		0.267			
Right	1.023	0.992‐1.054	0.143				1.018	0.986‐1.051	0.267			
Histology												
Squamous cell carcinoma	Ref		<0.001	Ref		<0.001	Ref		<0.001			
Adenocarcinoma	0.854	0.826‐0.884	<0.001	0.880	0.834‐0.930	<0.001	0.869	0.837‐0.901	<0.001			
Large cell carcinoma	1.129	1.027‐1.240	0.012	1.066	0.908‐1.252	0.432	1.172	1.061‐1.295	<0.001			
Others	1.108	1.066‐1.152	<0.001	0.960	0.893‐1.031	0.262	1.118	1.073‐1.166	<0.001			
T stage												
T1	Ref		<0.001	Ref		<0.001	Ref		<0.001	Ref		<0.001
T2	1.319	1.247‐1.395	<0.001	1.065	0.976‐1.161	0.155	1.357	1.277‐1.443	<0.001	1.076	0.979‐1.183	0.128
T3	1.569	1.461‐1.684	<0.001	1.262	1.126‐1.413	<0.001	1.676	1.553‐1.809	<0.001	1.324	1.172‐1.496	<0.001
T4	1.904	1.804‐2.003	<0.001	1.347	1.230‐1.476	<0.001	2.005	1.894‐2.121	<0.001	1.399	1.267‐1.545	<0.001
N stage												
N0	Ref		<0.001	Ref		<0.001	Ref		<0.001	Ref		<0.001
N1	0.813	0.757‐0.872	<0.001	1.055	0.947‐1.174	0.330	0.826	0.766‐0.891	<0.001	1.062	0.946‐1.193	0.310
N2	0.853	0.820‐0.888	<0.001	1.275	1.180‐1.378	<0.001	0.866	0.829‐0.904	<0.001	1.308	1.204‐1.422	<0.001
N3	0.930	0.883‐0.980	0.007	1.327	1.200‐1.467	<0.001	0.970	0.917‐1.025	0.297	1.390	1.249‐1.547	<0.001
Stage												
IIIA	Ref		<0.001				Ref		<0.001			
IIIB	1.362	1.322‐1.403	<0.001				1.413	1.369‐1.459	<0.001			
Tumor size	1.008	1.007‐1.009	<0.001	1.006	1.005‐1.007	<0.001	1.009	1.008‐1.010	<0.001	1.007	1.006‐1.008	<0.001
Treatment												
Surgery	Ref		<0.001	Ref		<0.001	Ref		<0.001	Ref		<0.001
Chemoradiotherapy	1.525	1.447‐1.607	<0.001	1.309	1.220‐1.403	<0.001	1.607	1.518‐1.702	<0.001	1.364	1.265‐1.472	<0.001
Radiotherapy	2.626	2.473‐2.789	<0.001	2.104	1.931‐2.293	<0.001	2.698	2.528‐2.880	<0.001	2.156	1.964‐1.367	<0.001
Chemotherapy	2.201	2.075‐2.336	<0.001	1.956	1.786‐2.141	<0.001	2.397	2.249‐2.554	<0.001	2.096	1.903‐1.308	<0.001
Best supportive care	4.462	4.232‐4.704	<0.001	3.954	1.669‐4.262	<0.001	4.629	4.370‐4.903	<0.001	4.106	3.785‐1.454	<0.001

95% CI, 95% confidence interval; HR, hazard ratio.

### 3.5 Construction and validation of nomogram

**Figure 4:**
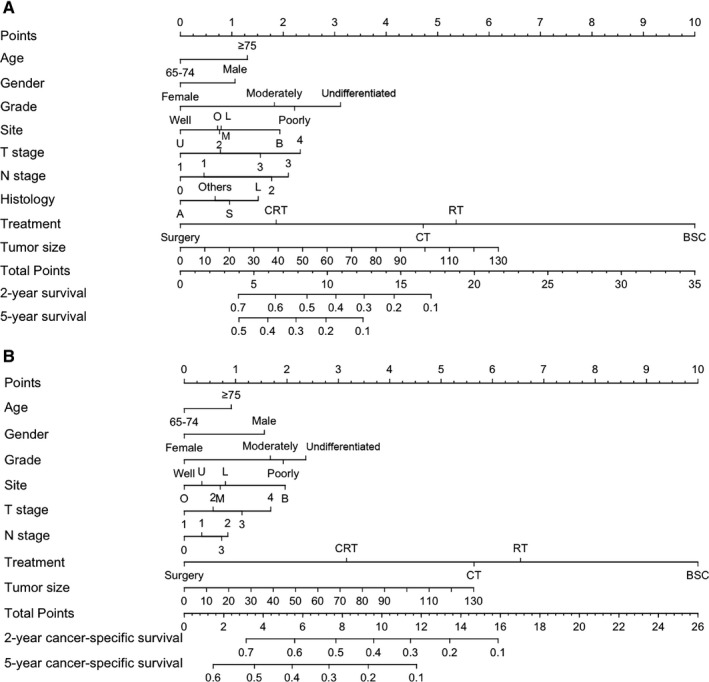
Nomogram predicting the 2‐ and 5‐year OS and CSS for elderly patients diagnosed with locally advanced nonsmall‐cell lung cancer (LA‐NSCLC). (A) Nomogram, including Age, Gender, Grade, Site, T stage, N stage, Histology, Treatment, and Tumor size for 2‐ and 5‐year OS in elderly patients with LA‐NSCLC; (B) Nomogram, including Age, Gender, Grade, Site, T stage, N stage, Treatment, and Tumor size for 2‐ and 5‐year CSS in elderly patients with LA‐NSCLC. Site: B, Main bronchus; L, Lower lobe; M, Middle lobe; O, Overlapping lesion of lung; U, Upper lobe. Histology: A, Adenocarcinoma; L, Large cell carcinoma; S, Squamous cell carcinoma. Treatment: BSC, Best Supportive Care; CRT, Chemoradiotherapy; CT, Chemotherapy; RT, Radiotherapy

Two prognostic nomograms for OS and CSS were firstly constructed based on the training set, which included all the significant independent factors by Cox proportional hazards regression analysis (Figure 4). By adding up and locating the scores on the total score scale, the estimated probability of OS and CSS at 2‐ and 5‐years could be determined.

**Figure 5:**
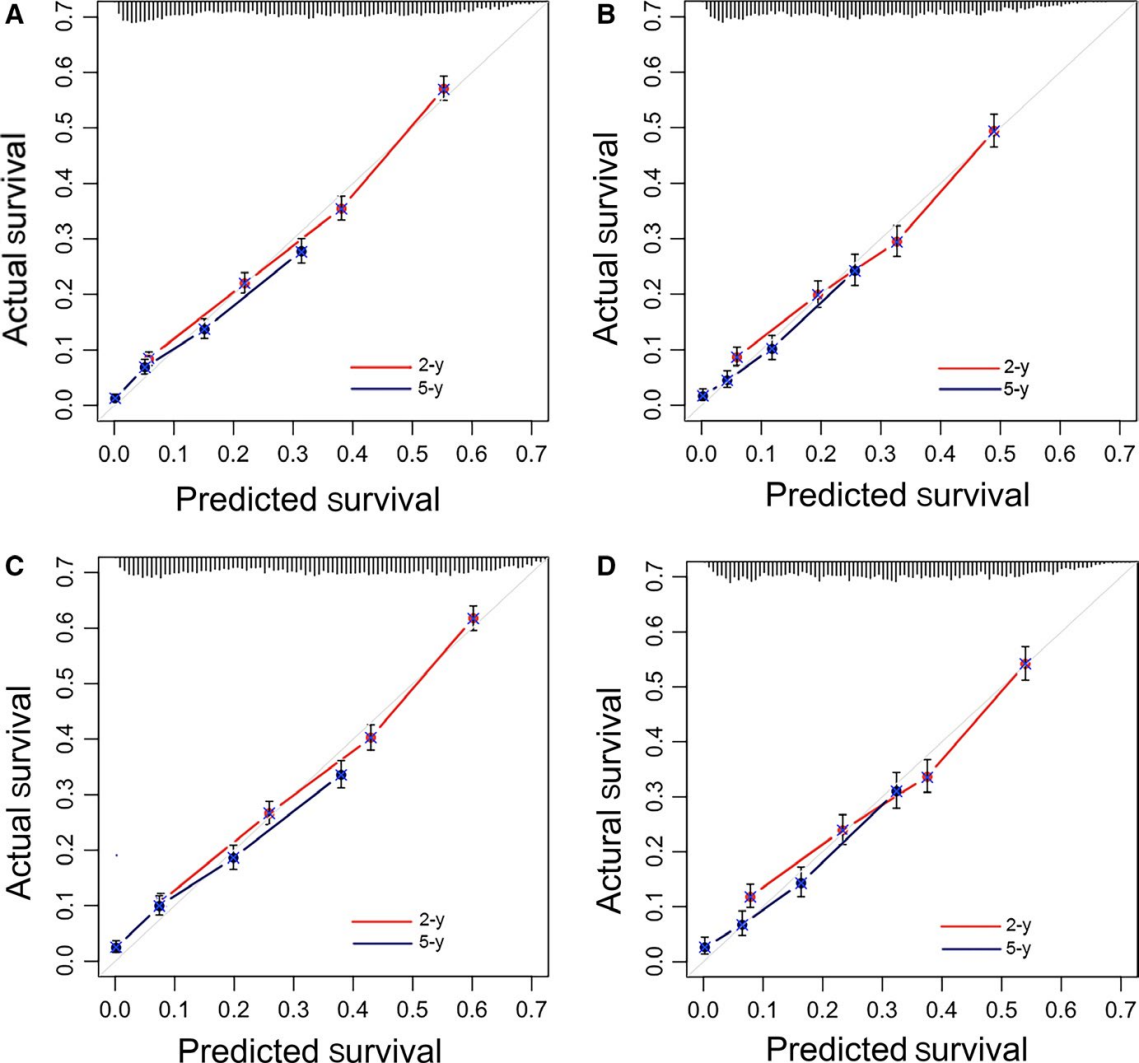
The calibration curves of nomograms for predicting OS and CSS in elderly patients with LA‐NSCLC using the training set and validation set. (A) Predicting 2‐ and 5‐year OS according to the training set; (B) Predicting 2‐ and 5‐year OS according to the validation set; (C) Predicting 2‐ and 5‐year CSS according to the training set; (D) Predicting 2‐ and 5‐year CSS according to the validation set. The x‐axis is nomogram‐predicted probability of survival and y‐axis is actual survival. The reference line is 45° and indicates perfect calibration

Nomograms were then externally validated in the validation set, C‐index values were used to measure discriminative ability. In the training cohort, the C‐index for the nomogram to predict OS and CSS were 0.692 (95% CI 0.678‐0.705) and 0.698 (95% CI 0.684‐0.712), respectively. Similarly, in the validation cohort, the C‐index for predicting OS and CSS were 0.674 (95% CI 0.654‐0.694) and 0.676 (95% CI 0.656‐0.696), respectively, demonstrating the accurate capacity in prognosis predicting. Importantly, the calibration plot showed that the predicted 2‐year and 5‐year OS and CSS closely correspond with the actual survival estimated by the Kaplan‐Meier method represented by the dotted lines in both the training set and validation set (Figure 5).

**Figure 6:**
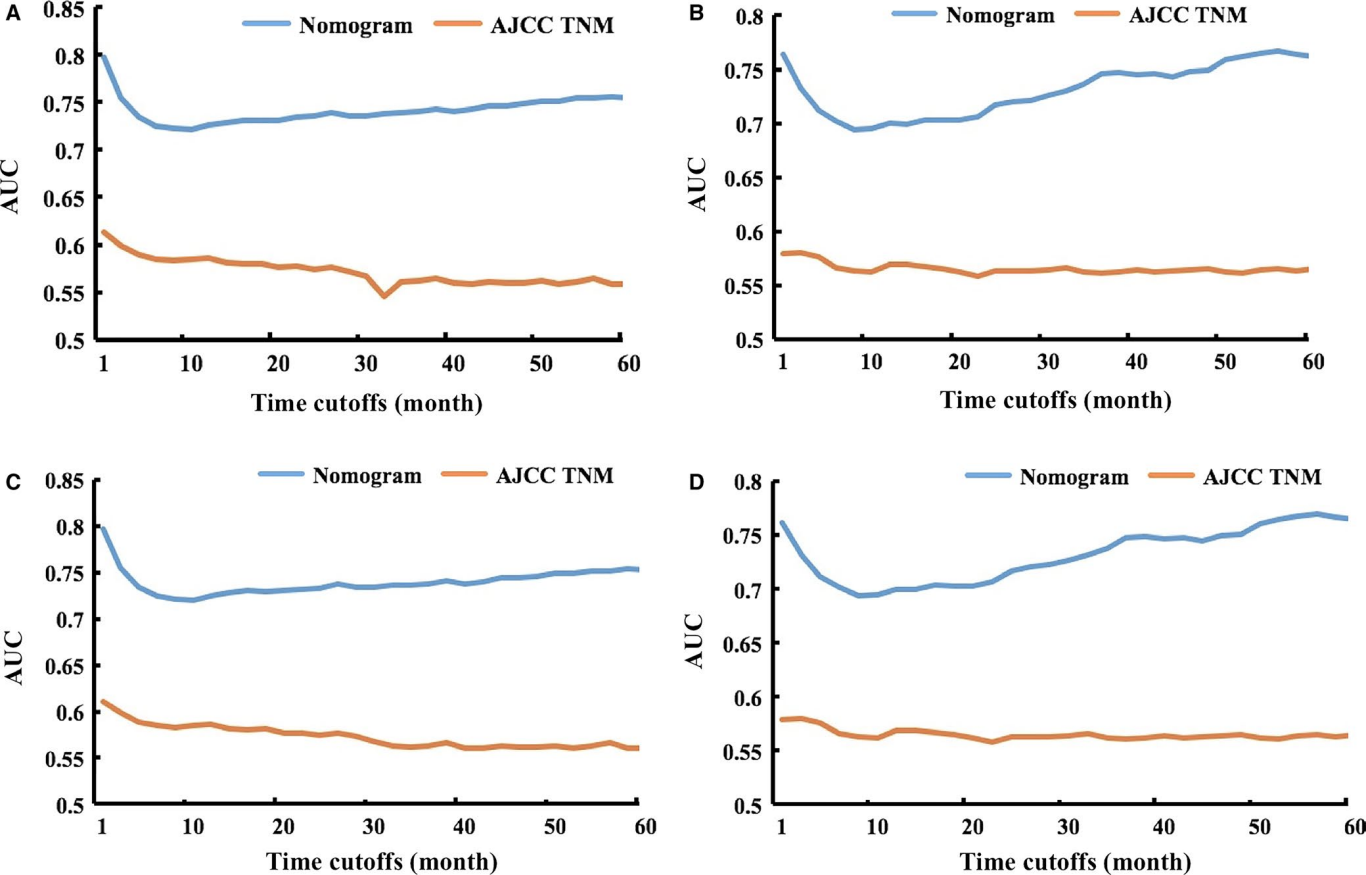
Area under the curve (AUC) models for comparing the predictive ability between nomograms and AJCC TNM stage. (A) The OS nomogram and AJCC TNM stage in the training set; (B) The OS nomogram and AJCC TNM stage in the validation set; (C) The CSS nomogram and AJCC TNM stage in the training set; (D) The CSS nomogram and AJCC TNM stage in the validation set. AUC was calculated for every month from the first to the 60th month

Furthermore, the AUC models were also constructed to compare the predictive ability between nomograms and AJCC TNM stage (Figure 6). Both in OS and CSS models, the integrated AUC for nomogram was superior to that of the seventh AJCC TNM classification (OS nomogram: 0.742 vs 0.572, *P* < 0.001 for training set and 0.731 vs 0.565, *P* < 0.001 for validation set; CSS nomogram: 0.740 vs 0.573, *P* < 0.001 for training set and 0.732 vs 0.565, *P* < 0.001 for validation set).

### 3.6 Subgroup analyses and sensitive analyses

Subgroup analysis of sex, tumor site, tumor grade, laterality, histology, T stage, N stage, AJCC TNM stage, and treatment was performed according to age group. A forest plot of HRs summarizing exploratory subgroup analyses found that in almost all subgroups, 65‐74 years age group subtype had better prognosis than ≥75 years age group (Figure S2). Only in radiotherapy subtype of treatment, HR showed no significant difference between two age groups (HR 0.98 95% CI 0.92‐1.06 *P* = 0.565). These results suggested that there were no significant confounders in this study.

Sensitive analysis was conducted to evaluate the impact of age for the prognosis for age more than 80 years (Table S2‐S4, Figure [Link], [Link]). It can be concluded that BSC was the most widely choice for patients ≥80 years while the selection of surgery was the least. But even in this older age group, surgery with or without (neo)adjuvant therapy still had best prognosis and BSC had worst prognosis, which is similar to the overall elderly population in our study. And the prognostic factors of patients aged over 80 years are also basically comparable to the general elderly population.

## 4 DISCUSSION

To our knowledge, this is the largest and newest population‐based study on the treatment patterns and their prognostic role in elderly LA‐NASCLC patients. Our study showed that the curative‐intent treatment options such as CRT and surgery are used in half of the patients aged 65‐74 years (56.4%); while 33.7% in patients aged ≥75 years. It seemed relatively conservative for the choose of aggressive treatments in elderly populations, compared to young patients, suggesting the worries of severe toxicities correlated with the increasing age in the real‐world practice. In consistent with the previous studies, the median OS and the 1/2/5 year OS rates in more elder patients aged ≥75 years were both inferior to that of patients aged 65‐74 years, and age was one of the independent risk factors for OS and CSS. Although it is hard to differentiate the cause of death from the cancer or the conditions related to age, such as more comorbidities, declined physiological function, and malnutrition; elder age was directly linked with the unfavorable prognosis, and was recommended to be taken into account in selection of treatment strategies due to the poor expected life.

However, the age may not be the only factor for determining the patient outcomes.^12^ Treatment patterns also independently predicted the prognosis in elderly LA‐NSCLC patients in our analysis. Although some studies showed that morbidity and postoperative mortality was positively associated with increasing age; elderly patients (≥70 years) considered the similar OS as younger patients undergoing surgery, and the higher operative rates were associated with increased survival in older patients with NSCLC (>66 years).^9^, ^13^, ^23^ In our present study, patients received surgery were found to have the best prognosis, which was statistically better than CRT, RT alone and chemotherapy alone in both age groups, also demonstrating the favorable role of surgery for elderly LA‐NSCLC patients. We proposed the benefits may be associated with the thorough assessment presurgery, the development of minimally invasive techniques, and the improvement in staging in this new era. It was not clear what subgroup of elderly LA‐NSCLC patients may get benefit from surgery; however, in any case, surgery may not be discarded in the selected patients as a positive option.

More and more evidence showed that aggressive CRT improved survival of elderly LA‐NSCLC compared to RT or chemotherapy alone.^15^, ^24^ Several retrospective studies and meta‐analysis demonstrated that patients older than 65 or 70 years could achieve longer OS of 12‐24 months when receiving CRT; and get OS of 7.6‐13.0 months for RT or chemotherapy alone.^15^, ^25^, ^26^, ^27^ More importantly, the randomized phase III trial by the Japan Clinical Oncology Group (JCOG 0301) reported that CRT prolonged OS compared to RT alone in 197 patients >70 years with a 32% reduction in risk of death.^28^ Hence, European Organization for Research and Treatment of Cancer (EORTC), the International Society of Geriatric Oncology (SIOG) and the expert panel in Spain all recommend CRT as the preferred treatment for elderly patients with unresectable LA‐NSCLC.^12^, ^29^ In agreement with the literature, our study found patients receiving CRT decreased the risk of death in both 65‐74 years and ≥75 years groups, and significantly prolonged OS compared with patients receiving BSC. However, combining chemotherapy and RT concurrently or sequentially remains controversial. Although concurrent CRT in elderly LA‐NSCLC patients was feasible and suggested the favorable survival in some studies; others showed that the superiority of concurrent CRT was not statistically significant versus sequential CRT, but with increase toxicity.^10^, ^25^, ^30^ Since the development of more tolerable chemotherapy regimens and the improvement of RT techniques, it will be helpful for determining the optimal schedules of CRT in the future studies. Nevertheless, elderly patients with LA‐NSCLC might be considered for sequential CRT when can't tolerate concurrent treatment with the risk of toxicities.

Except age and treatment patterns, patient gender, tumor site, size, histology, grade, and TNM stage were also found to be independent prognostic factors for elderly LA‐NSCLC patients. All of these important factors were integrated into concise nomogram models for predicting OS and CSS, respectively, which provide a practical tool for evaluating prognosis. Notably, these nomogram models were significantly more accurate than the AJCC TNM stage based on AUC analysis (OS nomogram: 0.742 vs 0.572, *P* < 0.001 for training set and 0.731 vs 0.565, *P* < 0.001 for validation set; CSS nomogram: 0.740 vs 0.573, *P* < 0.001 for training set and 0.732 vs 0.565, *P* < 0.001 for validation set); and calibration plots showed that the 2‐year, 5‐year survival rate predicted by our nomogram closely match the actual survival rate estimated by the Kaplan‐Meier method. More importantly, the identification ability and calibration ability of these nomograms were further confirmed in the external‐validation set. Based on the SEER database which consists of 18 cancer registries and covers almost thousands of hospitals and nearly 30% of the country's total population, we believed that these models representing a real‐world population were extremely reliable and convincible. Although more efforts with prospective trials still need to be made to prove our findings, our concise nomogram models may be feasible and valuable for individualized evaluating prognosis of elderly LA‐NSCLC patients and guiding the clinical treatment.

Deteriorated PS and more comorbidities were considered factors predicting worse prognosis in the elderly; however, information about PS, complication, and treatment tolerance were unavailable for evaluation in our study. The Comprehensive Geriatric Assessment (CGA), Fried frailty criteria, Triage Risk Screening Tool, and other instruments for assessing the status of elderly patients were recommended to be used combined with the prognostic nomograms in the future, which will include both the subjective and objective consideration for reaching the balance between survival and quality of life.^12^, ^31^, ^32^ Besides, treatment details were missing, such as number of lines that patients survived, the sequence of the treatment, the interactions existed between the treatments, treatment toxicity, cycle of chemotherapy, and radiation doses. These and other unmeasurable factors might affect the treatment option and the patient outcomes, which should be determined in future studies. Lastly, due to the retrospective nature of this study, potential of selection bias in treatment strategies can't be avoided, illustrated by the fact that patients with good performance and less comorbidities may receive more aggressive treatment.

## 5 CONCLUSION

Elderly patients of age 65‐74 years with LA‐NSCLC were more frequently treated with CRT and surgery, compared to patients aged ≥75 years who received relatively more BSC and RT alone. For all the elderly patients with LA‐NSCLC, the curative‐intent treatment (surgery or CRT) conferred better survival compared to chemotherapy alone, RT alone and BSC. Based on the independent clinicopathological factors, practical nomograms were developed and accurately predicted the 2‐ and 5‐ year OS and CSS rates of these patients. They may be helpful and valuable for guiding individualized treatment for elderly patients with LA‐NSCLC.

## CONFLICT OF INTEREST

The authors declare no conflict of interest.

## AUTHOR CONTRIBUTIONS

Conceptualization, MG and LW; Methodology, BL; Software, MG and YX; Validation, MG, YY and SW; Formal Analysis, MG; Investigation, XS; Writing‐Original Draft Preparation, MG; Writing‐Review & Editing, LW; Supervision, JY.
