# Supplementary figures and images for: Delineating the pattern of treatment for elderly locally advanced NSCLC and predicting outcomes by a validated model: A SEER based analysis
Source: Cancer Med. 2019 Apr 3;8(5):2587–98. doi: 10.1002/cam4.2127 (PMC6537004; doi:10.1002/cam4.2127)

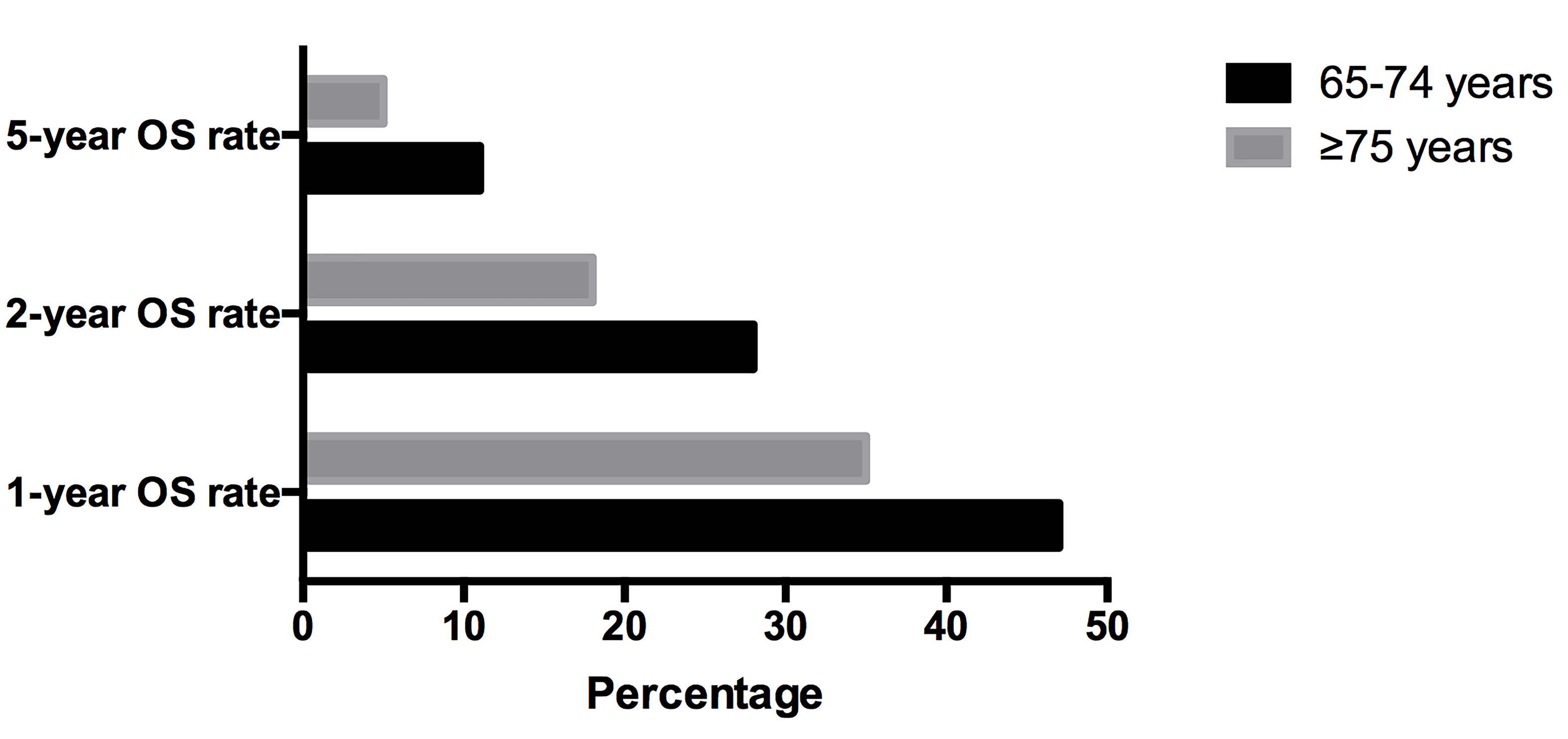

Supplement: Supplementary file 1 [file CAM4-8-2587-s001.tif]

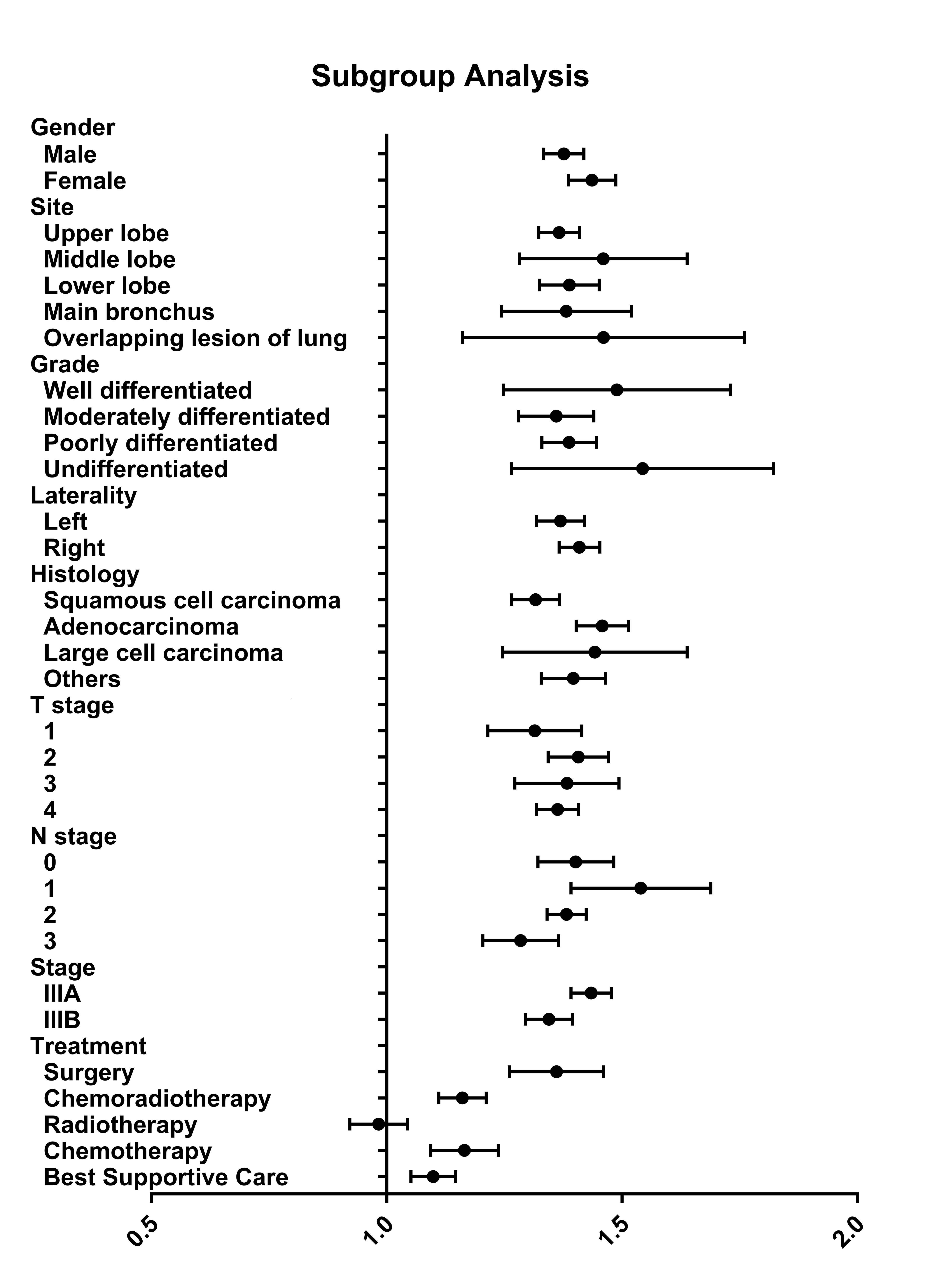

Supplement: Supplementary file 2 [file CAM4-8-2587-s002.tif]

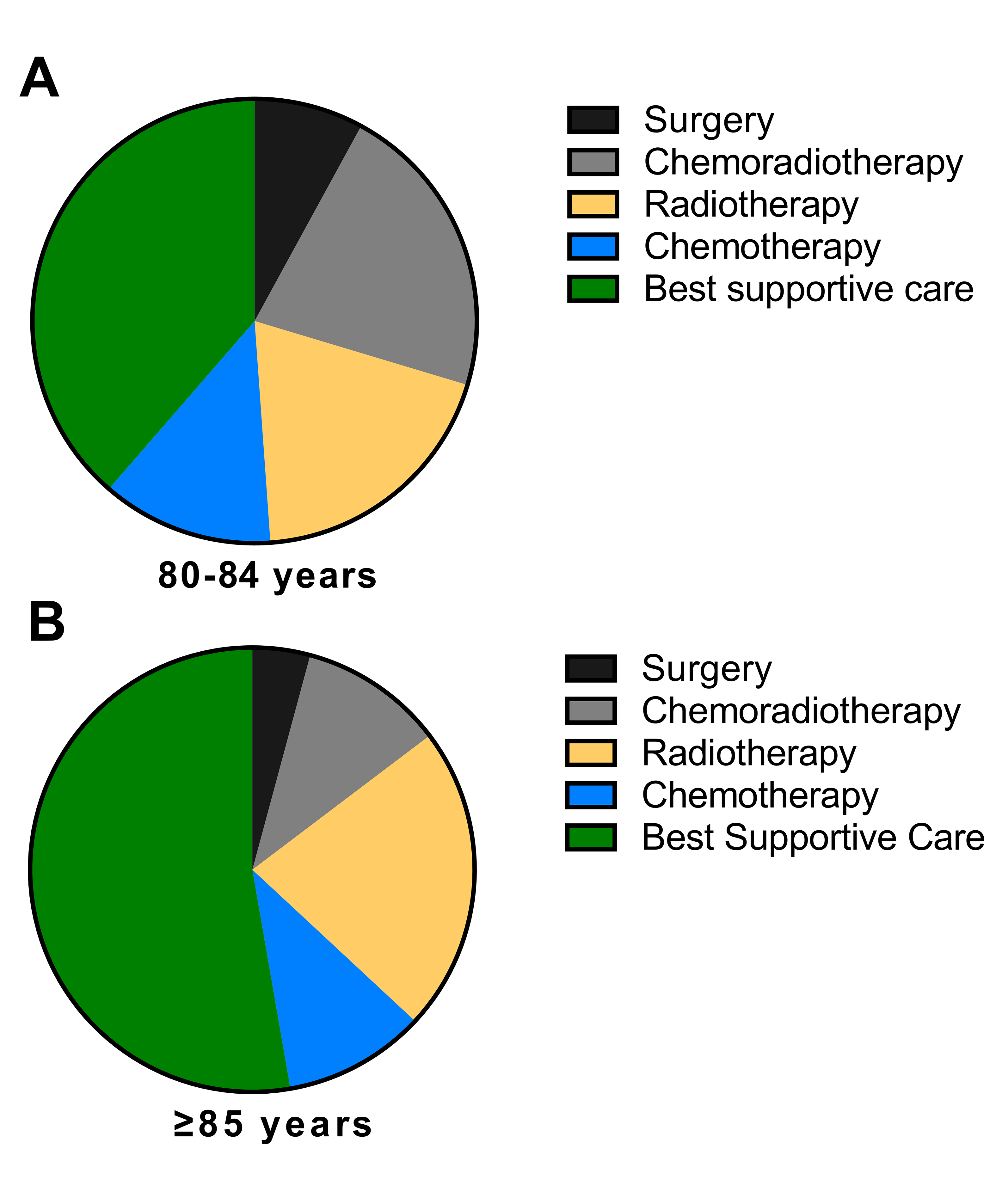

Supplement: Supplementary file 3 [file CAM4-8-2587-s003.tif]

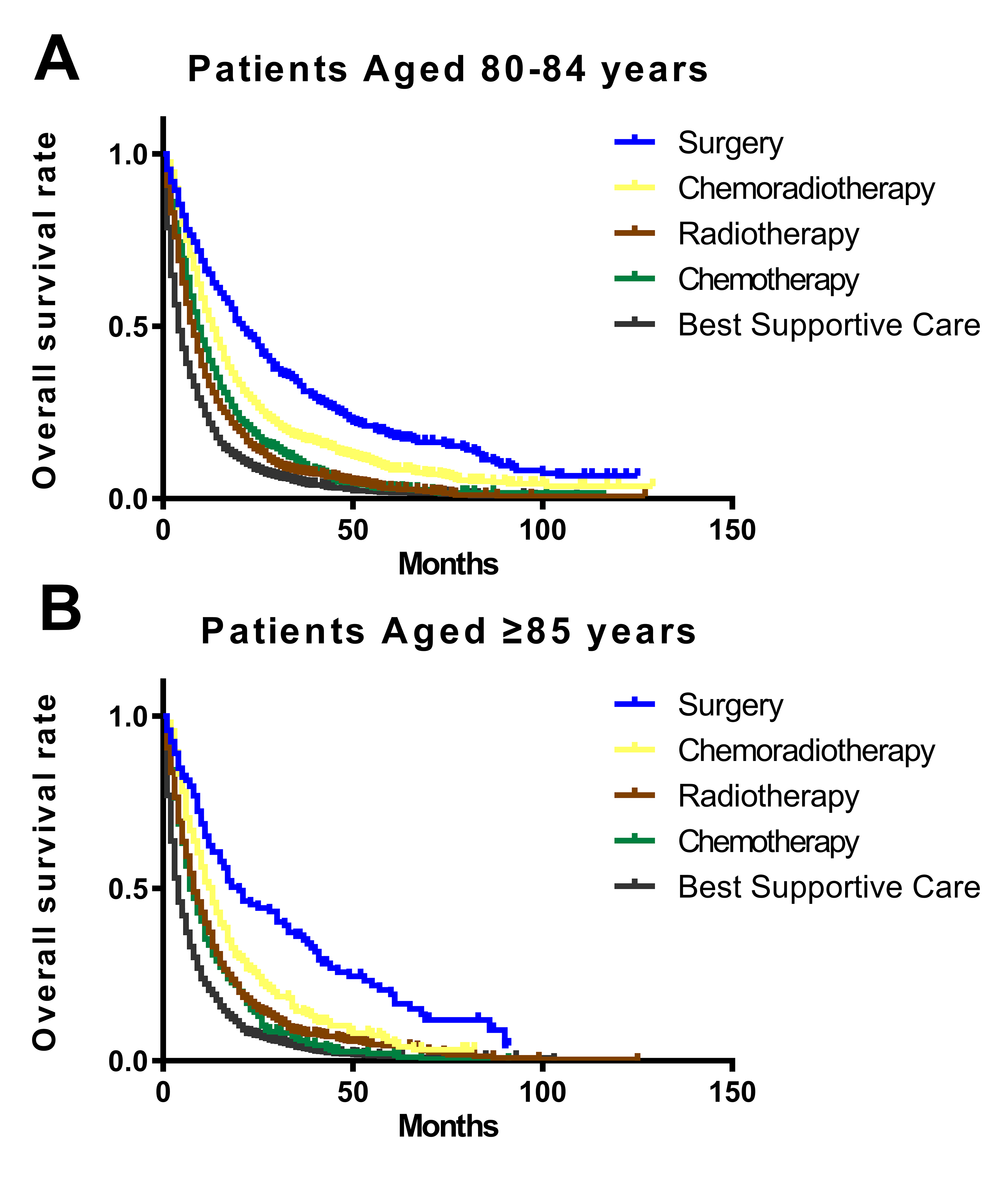

Supplement: Supplementary file 4 [file CAM4-8-2587-s004.tif]
