# Supplementary material for: Delineating the pattern of treatment for elderly locally advanced NSCLC and predicting outcomes by a validated model: A SEER based analysis
Source: Cancer Med. 2019 Apr 3;8(5):2587–98. doi: 10.1002/cam4.2127 (PMC6537004; doi:10.1002/cam4.2127)
Supplement: Supplementary file 5 [file CAM4-8-2587-s005.docx]

|  | **65-74 years** | | |  | **≥75 years** | | |
| --- | --- | --- | --- | --- | --- | --- | --- |
|  | **HR** | **CI** | **P value** |  | **HR** | **CI** | **P value** |
| Treatment |  |  | <0.001 |  |  |  | <0.001 |
| Surgery | Ref |  |  |  | Ref |  |  |
| Chemoradiotherapy | 1.554 | 1.469-1.644 | <0.001 |  | 1.272 | 1.184-1.367 | <0.001 |
| Radiotherapy | 2.827 | 2.623-3.048 | <0.001 |  | 1.973 | 1.830-2.126 | <0.001 |
| Chemotherapy | 2.221 | 2.071-2.383 | <0.001 |  | 1.791 | 1.651-1.944 | <0.001 |
| Best supportive care | 4.308 | 4.037-4.596 | <0.001 |  | 3.478 | 3.243-3.731 | <0.001 |

**Table S1.** Adjusted hazard ratio for different treatment in elderly patients according age groups.

*Abbreviations: HR, Hazard Ratio; 95%CI, 95% Confidence Interval.

|  |  | **Training set (n=5935)** | | | | | | |  | **Validation set (n=2658)** | | | | | | |
| --- | --- | --- | --- | --- | --- | --- | --- | --- | --- | --- | --- | --- | --- | --- | --- | --- |
|  |  | **80-84** | |  | **≥85** | |  | **P value** |  | **80-84** | |  | **≥85** | |  | **P value** |
|  |  | **No.** | **%** |  | **No.** | **%** |  |  |  | **No.** | **%** |  | **No.** | **%** |  |  |
| Gender |  |  |  |  |  |  |  | <0.001 |  |  |  |  |  |  |  | 0.269 |
| Male |  | 1936 | 52.1 |  | 1041 | 47.0 |  |  |  | 944 | 53.9 |  | 469 | 51.7 |  |  |
| Female |  | 1782 | 47.9 |  | 1176 | 53.0 |  |  |  | 806 | 46.1 |  | 439 | 48.3 |  |  |
| Site |  |  |  |  |  |  |  | 0.172 |  |  |  |  |  |  |  | 0.914 |
| Upper lobe |  | 1898 | 57.3 |  | 1103 | 57.8 |  |  |  | 924 | 59.1 |  | 455 | 59.5 |  |  |
| Middle lobe |  | 157 | 4.7 |  | 67 | 3.5 |  |  |  | 66 | 4.2 |  | 34 | 4.4 |  |  |
| Lower lobe |  | 1078 | 32.5 |  | 617 | 32.4 |  |  |  | 488 | 31.2 |  | 230 | 30.1 |  |  |
| Main bronchus |  | 147 | 4.4 |  | 93 | 4.9 |  |  |  | 71 | 4.5 |  | 40 | 5.2 |  |  |
| Overlapping lesion of lung |  | 34 | 1.0 |  | 27 | 1.4 |  |  |  | 15 | 1.0 |  | 6 | 0.8 |  |  |
| Grade |  |  |  |  |  |  |  | 0.290 |  |  |  |  |  |  |  | 0.774 |
| Well differentiated |  | 88 | 4.9 |  | 62 | 6.4 |  |  |  | 43 | 5.2 |  | 19 | 4.9 |  |  |
| Moderately differentiated |  | 560 | 30.9 |  | 292 | 30.1 |  |  |  | 253 | 30.6 |  | 117 | 30.1 |  |  |
| Poorly differentiated |  | 1109 | 61.3 |  | 581 | 60.0 |  |  |  | 511 | 61.7 |  | 239 | 61.4 |  |  |
| Undifferentiated |  | 53 | 2.9 |  | 34 | 3.5 |  |  |  | 21 | 2.5 |  | 14 | 3.6 |  |  |
| Laterality |  |  |  |  |  |  |  | 0.452 |  |  |  |  |  |  |  | 0.313 |
| Left |  | 1475 | 40.3 |  | 896 | 41.3 |  |  |  | 703 | 40.8 |  | 378 | 42.8 |  |  |
| Right |  | 2181 | 59.7 |  | 1271 | 58.7 |  |  |  | 1022 | 59.2 |  | 505 | 57.2 |  |  |
| Histology |  |  |  |  |  |  |  | 0.002 |  |  |  |  |  |  |  | 0.010 |
| Squamous cell carcinoma |  | 1255 | 33.8 |  | 644 | 29.0 |  |  |  | 696 | 39.8 |  | 315 | 34.7 |  |  |
| Adenocarcinoma |  | 1514 | 40.7 |  | 967 | 43.6 |  |  |  | 647 | 37.0 |  | 382 | 42.1 |  |  |
| Large cell carcinoma |  | 89 | 2.4 |  | 64 | 2.9 |  |  |  | 48 | 2.7 |  | 36 | 4.0 |  |  |
| Others |  | 860 | 23.1 |  | 542 | 24.4 |  |  |  | 359 | 20.5 |  | 175 | 19.3 |  |  |
| T stage |  |  |  |  |  |  |  | <0.001 |  |  |  |  |  |  |  | 0.001 |
| T1 |  | 365 | 10.3 |  | 168 | 7.9 |  |  |  | 166 | 10.0 |  | 62 | 7.1 |  |  |
| T2 |  | 961 | 27.1 |  | 534 | 25.3 |  |  |  | 478 | 28.7 |  | 217 | 24.7 |  |  |
| T3 |  | 278 | 7.8 |  | 115 | 5.4 |  |  |  | 146 | 8.8 |  | 65 | 7.4 |  |  |
| T4 |  | 1942 | 54.8 |  | 1297 | 61.4 |  |  |  | 876 | 52.6 |  | 535 | 60.9 |  |  |
| N stage |  |  |  |  |  |  |  | 0.003 |  |  |  |  |  |  |  | 0.001 |
| N0 |  | 724 | 20.1 |  | 507 | 24.1 |  |  |  | 328 | 19.3 |  | 227 | 26.2 |  |  |
| N1 |  | 203 | 5.6 |  | 122 | 5.8 |  |  |  | 110 | 6.5 |  | 51 | 5.9 |  |  |
| N2 |  | 2215 | 61.4 |  | 1227 | 58.4 |  |  |  | 1055 | 61.9 |  | 499 | 57.6 |  |  |
| N3 |  | 467 | 12.9 |  | 246 | 11.7 |  |  |  | 210 | 12.3 |  | 89 | 10.3 |  |  |
| Stage |  |  |  |  |  |  |  | 0.250 |  |  |  |  |  |  |  | 0.790 |
| IIIA |  | 2343 | 63.0 |  | 1364 | 61.5 |  |  |  | 1127 | 64.4 |  | 580 | 63.9 |  |  |
| IIIB |  | 1375 | 37.0 |  | 853 | 38.5 |  |  |  | 623 | 35.6 |  | 328 | 36.1 |  |  |
| Tumor size |  | / | / |  | / | / |  | 0.293 |  | / | / |  | / | / |  | 0.542 |
| Treatment |  |  |  |  |  |  |  | <0.001 |  |  |  |  |  |  |  | <0.001 |
| Surgery |  | 307 | 8.3 |  | 99 | 4.5 |  |  |  | 128 | 7.3 |  | 32 | 3.5 |  |  |
| Chemoradiotherapy |  | 781 | 21.0 |  | 224 | 10.1 |  |  |  | 405 | 23.1 |  | 104 | 11.5 |  |  |
| Radiotherapy |  | 695 | 18.7 |  | 470 | 21.2 |  |  |  | 356 | 20.3 |  | 225 | 24.8 |  |  |
| Chemotherapy |  | 469 | 12.6 |  | 254 | 11.5 |  |  |  | 215 | 12.3 |  | 69 | 7.6 |  |  |
| Best supportive care |  | 1466 | 39.4 |  | 1170 | 52.8 |  |  |  | 646 | 36.9 |  | 478 | 52.6 |  |  |

**Table S2.** The demographic and clinicopathological variables of the training set and validation set of patients ≥80 years.

*The P values were determined by the χ2-test for categorical variables and the one-way ANOVA (Analysis of Variance) for continuous variables. /: not applicable.

|  | **80-84 years** | | |  | **≥85 years** | | |
| --- | --- | --- | --- | --- | --- | --- | --- |
|  | **HR** | **CI** | **P value** |  | **HR** | **CI** | **P value** |
| Treatment |  |  | <0.001 |  |  |  | <0.001 |
| Surgery | Ref |  |  |  | Ref |  |  |
| Chemoradiotherapy | 1.282 | 1.126-1.459 | <0.001 |  | 1.340 | 1.054-1.702 | <0.001 |
| Radiotherapy | 2.019 | 1.774-2.299 | <0.001 |  | 1.819 | 1.460-2.266 | <0.001 |
| Chemotherapy | 1.687 | 1.463-1.946 | <0.001 |  | 1.822 | 1.427-2.326 | <0.001 |
| Best supportive care | 3.326 | 2.941-3.762 | <0.001 |  | 3.214 | 2.599-3.975 | <0.001 |

**Table S3.** Adjusted hazard ratio for different treatment in patients ≥80 years according age groups.

*Abbreviations: HR, Hazard Ratio; 95%CI, 95% Confidence Interval.

|  | **OS** | | | | | | |  | **CSS** | | | | | | |
| --- | --- | --- | --- | --- | --- | --- | --- | --- | --- | --- | --- | --- | --- | --- | --- |
|  | **Univariate analysis** | | |  | **Multivariate analysis** | | |  | **Univariate analysis** | | |  | **Multivariate analysis** | | |
|  | **HR** | **CI** | **P value** |  | **HR** | **CI** | **P value** |  | **HR** | **CI** | **P value** |  | **HR** | **CI** | **P value** |
| Age |  |  | <0.001 |  |  |  |  |  |  |  | <0.001 |  |  |  |  |
| 80-84 | Ref |  |  |  |  |  |  |  | Ref |  |  |  |  |  |  |
| ≥85 | 1.420 | 1.379-1.462 | <0.001 |  |  |  |  |  | 1.196 | 1.127-1.268 | <0.001 |  |  |  |  |
| Gender |  |  | <0.001 |  |  |  | <0.001 |  |  |  | 0.008 |  |  |  | 0.005 |
| Male | Ref |  |  |  | Ref |  |  |  | Ref |  |  |  | Ref |  |  |
| Female | 0.908 | 0.861-0.958 | <0.001 |  | 0.860 |  | <0.001 |  | 0.925 | 0.874-0.980 | 0.008 |  | 0.869 | 0.787-0.959 | 0.005 |
| Site |  |  | <0.001 |  |  |  | 0.008 |  |  |  | <0.001 |  |  |  | 0.018 |
| Upper lobe | Ref |  |  |  | Ref |  |  |  | Ref |  |  |  | Ref |  |  |
| Middle lobe | 1.121 | 0.971-1.295 | 0.118 |  | 1.268 | 1.016-1.583 | 0.036 |  | 1.101 | 0.944-1.285 | 0.219 |  | 1.267 | 1.000-1.604 | 0.05 |
| Lower lobe | 1.143 | 1.073-1.217 | <0.001 |  | 1.134 | 1.025-1.254 | 0.015 |  | 1.129 | 1.056-1.207 | <0.001 |  | 1.117 | 1.002-1.244 | 0.046 |
| Main bronchus | 1.639 | 1.433-1.875 | <0.001 |  | 1.369 | 1.070-1.753 | 0.013 |  | 1.621 | 1.405-1.871 | <0.001 |  | 1.396 | 1.078-1.808 | 0.012 |
| Overlapping lesion of lung | 1.298 | 0.998-1.688 | 0.051 |  | 0.962 | 0.646-1.433 | 0.849 |  | 1.256 | 0.947-1.667 | 0.114 |  | 0.944 | 0.618-1.443 | 0.79 |
| Grade |  |  | <0.001 |  |  |  | 0.038 |  |  |  | <0.001 |  |  |  | 0.001 |
| Well differentiated | Ref |  |  |  | Ref |  |  |  | Ref |  |  |  | Ref |  |  |
| Moderately differentiated | 1.099 | 0.910-1.326 | 0.327 |  | 1.246 | 0.994-1.562 | 0.057 |  | 1.107 | 0.903-1.357 | 0.328 |  | 1.297 | 1.016-1.655 | 0.037 |
| Poorly differentiated | 1.299 | 1.084-1.557 | 0.005 |  | 1.354 | 1.083-1.692 | 0.008 |  | 1.336 | 1.098-1.626 | 0.004 |  | 1.476 | 1.165-1.871 | 0.001 |
| Undifferentiated | 1.780 | 1.349-2.348 | <0.001 |  | 1.438 | 1.003-2.062 | 0.048 |  | 1.917 | 1.430-2.568 | <0.001 |  | 1.755 | 1.228-2.507 | 0.002 |
| Laterality |  |  | 0.116 |  |  |  |  |  |  |  | 0.186 |  |  |  |  |
| Left | Ref |  |  |  |  |  |  |  | Ref |  |  |  |  |  |  |
| Right | 1.045 | 0.989-1.104 | 0.116 |  |  |  |  |  | 1.040 | 0.981-1.103 | 0.186 |  |  |  |  |
| Histology |  |  | <0.001 |  |  |  | 0.040 |  |  |  | <0.001 |  |  |  |  |
| Squamous cell carcinoma | Ref |  |  |  | Ref |  |  |  | Ref |  |  |  |  |  |  |
| Adenocarcinoma | 0.940 | 0.882-1.001 | 0.056 |  | 0.896 | 0.803-0.998 | 0.047 |  | 0.971 | 0.908-1.039 | 0.397 |  |  |  |  |
| Large cell carcinoma | 1.253 | 1.059-1.482 | 0.009 |  | 1.273 | 0.959-1.690 | 0.095 |  | 1.306 | 1.093-1.559 | 0.003 |  |  |  |  |
| Others | 1.146 | 1.067-1.231 | <0.001 |  | 1.005 | 0.876-1.154 | 0.942 |  | 1.181 | 1.095-1.276 | <0.001 |  |  |  |  |
| T stage |  |  | <0.001 |  |  |  |  |  |  |  | <0.001 |  |  |  |  |
| T1 | Ref |  |  |  |  |  |  |  | Ref |  |  |  |  |  |  |
| T2 | 1.320 | 1.184-1.472 | <0.001 |  |  |  |  |  | 1.333 | 1.185-1.500 | <0.001 |  |  |  |  |
| T3 | 1.554 | 1.350-1.788 | <0.001 |  |  |  |  |  | 1.646 | 1.417-1.912 | <0.001 |  |  |  |  |
| T4 | 1.903 | 1.720-2.104 | <0.001 |  |  |  |  |  | 1.965 | 1.762-2.191 | <0.001 |  |  |  |  |
| N stage |  |  | <0.001 |  |  |  |  |  |  |  | 0.009 |  |  |  |  |
| N0 | Ref |  |  |  |  |  |  |  | Ref |  |  |  |  |  |  |
| N1 | 0.897 | 0.790-1.019 | 0.095 |  |  |  |  |  | 0.890 | 0.776-1.021 | 0.098 |  |  |  |  |
| N2 | 0.864 | 0.808-0.924 | <0.001 |  |  |  |  |  | 0.884 | 0.822-0.950 | 0.001 |  |  |  |  |
| N3 | 0.853 | 0.775-0.940 | 0.001 |  |  |  |  |  | 0.889 | 0.802-0.985 | 0.024 |  |  |  |  |
| Stage |  |  | <0.001 |  |  |  | 0.068 |  |  |  | <0.001 |  |  |  | 0.024 |
| IIIA | Ref |  |  |  | Ref |  |  |  | Ref |  |  |  | Ref |  |  |
| IIIB | 1.289 | 1.220-1.362 | <0.001 |  | 1.098 | 1.001-1.215 | 0.068 |  | 1.340 | 1.264-1.421 | <0.001 |  | 1.131 | 1.016-1.258 | 0.024 |
| Tumor size | 1.009 | 1.008-1.010 | <0.001 |  | 1.011 | 1.009-1.013 | <0.001 |  | 1.010 | 1.008-1.011 | <0.001 |  | 1.012 | 1.010-1.014 | <0.001 |
| Treatment |  |  | <0.001 |  |  |  | <0.001 |  |  |  | <0.001 |  |  |  | <0.001 |
| Surgery | Ref |  |  |  | Ref |  |  |  | Ref |  |  |  | Ref |  |  |
| Chemoradiotherapy | 1.333 | 1.170-1.519 | <0.001 |  | 1.067 | 0.899-1.266 | 0.458 |  | 1.456 | 1.260-1.682 | <0.001 |  | 1.171 | 0.974-1.408 | 0.092 |
| Radiotherapy | 1.980 | 1.744-2.249 | <0.001 |  | 1.664 | 1.411-1.963 | <0.001 |  | 2.075 | 1.801-2.389 | <0.001 |  | 1.774 | 1.482-2.123 | <0.001 |
| Chemotherapy | 1.929 | 1.685-2.209 | <0.001 |  | 1.781 | 1.467-2.161 | <0.001 |  | 2.151 | 1.854-2.495 | <0.001 |  | 1.95 | 1.582-2.403 | <0.001 |
| Best supportive care | 3.568 | 3.166-4.021 | <0.001 |  | 3.310 | 2.840-3.859 | <0.001 |  | 3.878 | 3.396-4.430 | <0.001 |  | 3.65 | 3.087-4.316 | <0.001 |

**Table S4.** Univariate and multivariate survival analyses of OS and CSS in patients ≥80 years with LA-NSCLC.

*Abbreviations: HR, Hazard Ratio; 95%CI, 95% Confidence Interval.
